# 3D echocardiography derived reference values and determinants of left ventricular twist and torsion from the population-based STAAB cohort study

**DOI:** 10.1038/s41598-024-81662-x

**Published:** 2025-02-06

**Authors:** Julia Napp, Götz Gelbrich, Floran Sahiti, Vladimir Cejka, Dora Pelin, Lena Schmidbauer, Mengmeng Chen, Niklas Hitschrich, Marcus Schreckenberg, Stefan Frantz, Peter U. Heuschmann, Stefan Störk, Caroline Morbach

**Affiliations:** 1 Academic Core Lab Ultrasound-Based Cardiovascular Imaging, Department Clinical Research and Epidemiology, Comprehensive Heart Failure Center, University Hospital Würzburg, Am Schwarzenberg 15, 97078 Würzburg, Germany; 2 Department of Medicine I, University Hospital Würzburg, Würzburg, Germany; 3 Institute of Clinical Epidemiology and Biometry, University of Würzburg, Würzburg, Germany; 4 Clinical Trial Center, University Hospital Würzburg, Würzburg, Germany; 5 Institute for Medical Data Sciences, University Hospital Würzburg, Würzburg, Germany; 6 TOMTEC Imaging Systems GmbH, Unterschleißheim, Germany

**Keywords:** 3D echocardiography, Left ventricle, Twist, Torsion, Population-based cohort, Cardiology, Heart failure

## Abstract

Left ventricular (LV) rotational function parameters provide in-depth information about LV mechanical function as well as prognostic information. Using three-dimensional (3D) echocardiography, we identified determinants of LV “twist” and “torsion”, and established reference values using a large population-based cohort. 3D echocardiography images were recorded in n = 2803 subjects within the prospective STAAB cohort study investigating a representative age- and sex-stratified sample of residents of the city of Würzburg, aged 30–79 years, without history of heart failure. Valid 3D image analysis was performed in n = 1831 (65.3%) subjects (mean age 57 ± 11 years, 49.3% women). Using general linear models, we identified determinants of LV twist and torsion: there was a positive association between LV rotational parameters and age, female sex, and blood pressure but a negative association with body weight. From a subset of 479 apparently healthy individuals exhibiting no cardiovascular (CV) risk factors or CV disease (mean age 52 ± 10 years, 56.4% women), we derived reference percentiles for twist and torsion. LV rotation is determined by a complex interplay of sub-endocardial and sub-epicardial fibers which might be affected differentially by potential risk factors. We found a differential association with respective determinants as LV rotational parameters increased with age and with higher blood pressure but decreased with higher body weight. Further research is needed to elucidate these associations in more detail and to determine the additional information contributed by twist and torsion. To facilitate respective attempts and to set an individual’s results in relation to a population-based reference, we derived normal values for twist and torsion from a sub-collective of healthy individuals.

## Introduction

**Fig. 1:**
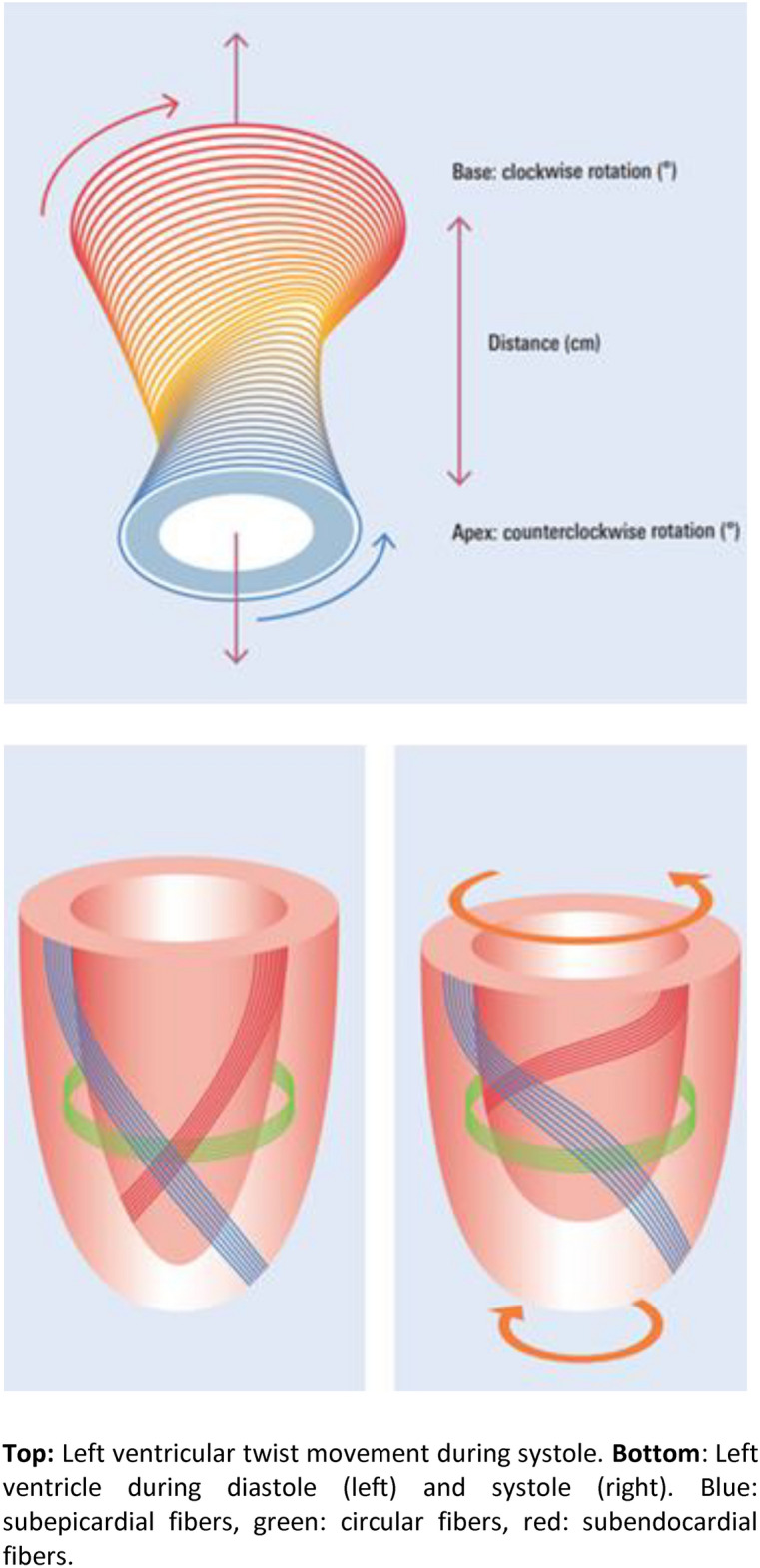
Schematic representation of the left ventricular rotation during a cardiac cycle. Top: Left ventricular twist movement during systole. Bottom: Left ventricle during diastole (left) and systole (right). Blue: subepicardial fibers, green: circular fibers, red: subendocardial fibers.

The left ventricular (LV) rotational parameters twist and torsion provide important information about the mechanical functionality of the LV. Viewed from the cardiac apex, the base of the heart rotates clockwise during systole, while the apex rotates counterclockwise (Fig. 1). LV twist (measured in °) describes the sum of the absolute values of the maximum apical and basal angle of rotation^1^. Torsion (measured in °/cm) is the twist normalized to the measured distance between apex and base^2^.

Prior observational studies consistently reported on the prognostic relevance of LV rotational parameters in various cardiovascular conditions. E.g., in subjects with non-ischemic dilated cardiomyopathy (DCM), reduced torsion accurately identified patients with a reduced functional capacity and was associated with more frequent hospitalization when compared to patients with preserved torsion^3,4^. In patients after ST-elevation myocardial infarction, adding LV torsion to conventional prognostic factors such as left ventricular ejection fraction (LVEF) and infarct size, resulted in improved risk classification^5^. Furthermore, twist and torsion predicted the risk of two-year all-cause death in patients with severe aortic stenosis after transcatheter aortic valve implantation^6^. 

Three-dimensional echocardiography (3DE) offers the possibility of reliably measuring twist and torsion. This has been validated using sonomicrometry in animal studies^7,8^ and cardiac magnetic resonance imaging (MRI) in human studies as a reference standard^9^. There, torsion consistently increased with age^9–13^. Previously published reference values for 3D measured twist and torsion varied widely, most likely since they were derived from heterogeneous collectives and convenience samples^9,12–15^.

Our aim was to establish 3DE-derived reference values that account for the determinants of LV rotational function by using a large population-based cohort stratified for age and sex and covering a broad age range.

## Methods

### Study population and recruitment

Data were acquired as part of the *Characteristics and Course of Heart Failure Stages A-B and Determinants of Progression* (STAAB) cohort study. The study comprises a representative sample of residents of the city of Würzburg (Germany) without self-reported heart failure, aged 30–79 years, stratified for age (10:27:27:27:10 for the respective decades) and sex (1:1). Data for the analyses were obtained from the first follow-up examination, which took place from December 2017 to August 2021. The detailed study design and methodology have been published^16,17^. Since STAAB is not a clinical trial, it has not been registered and provided with a clinical trial number.

### Examination

In accordance with the standard operating procedures implemented for the STAAB cohort study, anthropometry and blood pressure were assessed^16^. The central laboratory at the University Hospital of Würzburg conducted the laboratory measurements from venous blood. Medical history and current pharmacotherapy were assessed by a trained physician, who also performed the physical examination^16,18^.

### Image acquisition and analysis

**Fig. 2:**
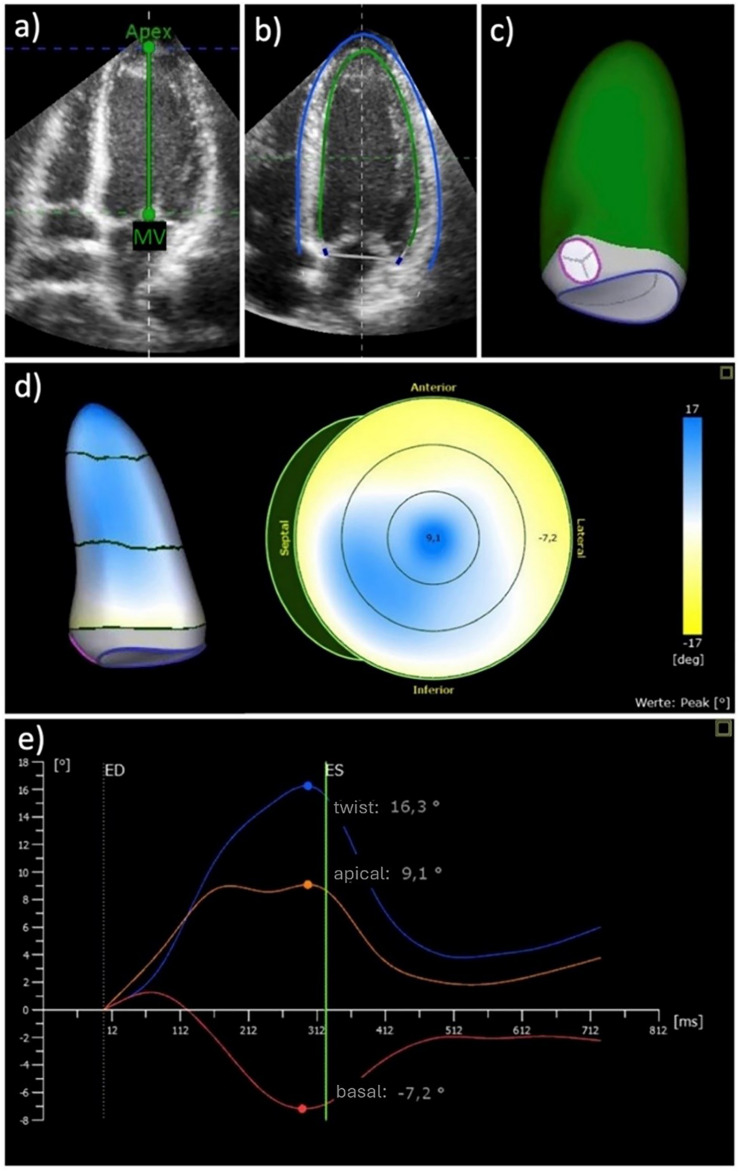
Image analysis and determination of left ventricular (LV) twist through 3D echocardiography. (**a**) Marking of the mitral valve (MV) and apex of the LV. (**b**) Contour of the LV (blue line), the endocardium (green line) and marking of the mitral valve (blue dots). (**c**) 3D reconstruction of the LV. (**d**) Visualisation of the apical (blue) and basal (yellow) rotation of the LV. (**e**) LV rotation during the cardiac cycle. Orange line: apical rotation, red line: basal rotation, ED: end-diastole, ES: end-systole. Twist (blue dot): the sum of the absolute values of the maximum apical (orange dot) and basal (red dot) rotation.

Transthoracic echocardiography was performed by trained and internally certified personnel using a Vivid E95 ultrasound scanner equipped with a M5SC-D 1.5-4.6 MHz transducer (GE Healthcare, Horten, Norway). The pre-specified protocol started with a comprehensive set of 2D images and Doppler measurements to characterize cardiac structure and function. A minimum of three cardiac cycles per angulation was recorded and stored digitally. The scan was completed by 3D images from the apical window focusing on covering the complete LV. For each view, we performed a single-beat and a multi-beat acquisition aiming for higher frame rates, respectively^16,19^. In the context of a cooperation project^20^, 3D images were analyzed off-line using custom software (TomtecArena®, Tomtec Imaging Systems, Unterschleißheim, Germany). For each subject, the 3D dataset with the best image quality was selected. Images not covering the complete LV, with poor image quality (e.g. low resolution, high attenuation, reverberation artifacts), or containing stitching artifacts were excluded from the analysis. With the start of the semi-automatic analysis, the views were first correctly aligned at the time points of end-diastole and end-systole. For this purpose, the mitral and aortic valves and the apex were marked in different views. The time at which the LV cavity was at its largest was marked as end-diastole, while the time at which the cavity was at its smallest was marked as end-systole. These marks were created automatically by the software but were checked by the observer and manually adjusted as needed. The 3D datasets then appeared as multi-plane reconstruction images, presented in three standard long-axis views (apical four-chamber, three-chamber, and two-chamber views) and a short-axis view. Contours of the endocardium and the location of the mitral and aortic valves were automatically marked by the software and adjusted by the examiner as necessary. The LV outflow tract, papillary muscles, and trabeculae were included in the LV cavity. Finally, the software reconstructed a beating LV so that the adjustments could be verified in three dimensions. LV volumes and functional parameters including twist and torsion as well as circumferential strain^21^ were then calculated automatically (Fig. 2).

No analysis was performed if there was no sufficient image quality for analysis, if the ventricle was not completely acquired, or if stitching artifacts were present.

### Collectives

Determinants of LV twist and torsion were derived from the total sample including all individuals with valid 3D LV analysis. To generate reference percentiles, we selected individuals who were considered “apparently healthy” because they met all of the following criteria: No cardiovascular disease (i.e., no previous myocardial infarction or stroke, coronary artery disease, peripheral artery disease); no hypertension (i.e., systolic blood pressure ≤ 139 mmHg^22^ and no anti-hypertensive pharmacotherapy; no dyslipidemia (i.e., low-density lipoprotein LDL ≤ 189 mg/dl^23^ and no lipid-lowering pharmacotherapy); body mass index (BMI) ≤ 30 kg/m^2,22^; no diabetes mellitus (i.e., glycosylated hemoglobin ≤ 6.5%, fasting plasma glucose ≤ 7.0 mmol/l and 2-h plasma glucose ≤ 11.1 mmol/l after glucose loading^24^ and no anti-diabetic medication); no current or former smoker.

### Data analysis

For statistical analysis, we used SPSS (Version 29, SPSS Inc., Chicago, USA). Data are presented as frequency (percent), mean (± standard deviation), or median (quartiles), as appropriate. Groups were compared using parametric or non-parametric tests, as appropriate. Inter- and intraobserver variability was tested using Bland–Altman plots. *P* values lower than 0.05 were considered statistically significant.

The factors associated with twist and torsion were determined using general linear models. In these models, age was reported sex-adjusted, while sex was reported in different models once adjusted for age and once adjusted for both age and height. All other variables were adjusted for sex and age. For each parameter, it was examined whether there was a significant interaction with sex (reference category: women). If there was a significant sex interaction, the respective model was additionally applied with men as the reference category. For the identification of associations of LV twist and torsion with volume and other functional parameters of the LV, 2D measured parameters were used apart from global circumferential strain (GCS), which was assessed only three-dimensionally. Aiming to identify potential determinants of LV rotation, we did not combine respective parameters with collinear echocardiographic parameters in the same statistical model.

To determine reference percentiles, each variable was tested for an interaction between sex and height, as well as sex and age. The Shapiro–Wilk test was used to check whether the parameters were normally distributed. The homogeneity of the residuals was examined using a histogram. Two variables were created, indicating the zero point of the constant term at a height of 170 cm (h170) and at the age of 50 years (a50). Women were chosen as the reference category. The constant term, as well as the deviation of the constant term for age, height, and male sex were presented and tested for significance. For non-normally distributed values, the deviation of the residuals from the constant term was reported at the 2.5th, 10th, 25th, 50th, 75th, 90th, and 97.5th percentiles.

## Results

### Study population and quality measures

**Fig. 3:**
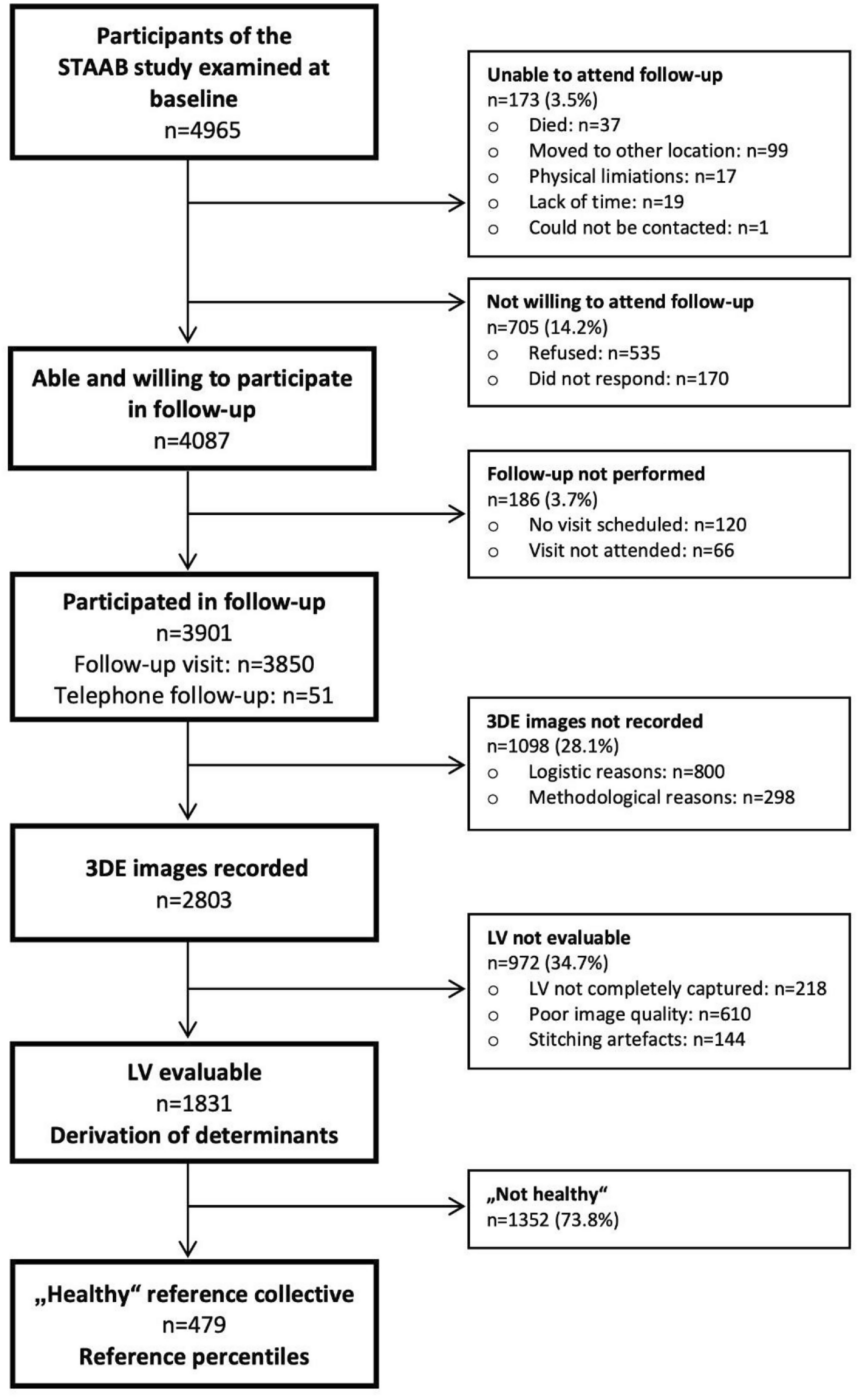
Study consort. 3DE: three-dimensional echocardiography, LV: left ventricle, STAAB: prospective *Characteristics and Course of Heart Failure Stages A-B and Determinants of Progression*. The follow-up status of STAAB participants examined at baseline refers to the end of observation for each participant, defined by the dates of follow-up, declaration of non-participation in follow-up by the participant, death, or August 19th, 2021 (the last follow-up was performed on this date), whatever occurred first. Those who did not respond were alive by August 19th, 2021 as confirmed by the residents’ registration office.

We recorded 3D images from 2803 participants (Fig. 3). Of those, 653 (23.3%) individuals were considered “apparently healthy”. Analysis of 3DE measurement was feasible in 1831 subjects (feasibility 65.3%; results of LV size and function are given in the additional Table 1). Within the healthy subsample (n = 479, mean age 52 years, 56.4% women) the feasibility was higher (73.5%). Subjects with evaluable LV were significantly younger, had lower BMI and a more favorable risk profile as well as more favorable cardiac structure and function (Table 1). Intra- and interobserver reproducibility was excellent for both twist and torsion (see additional Figs. 1, 2).

**Table 1:** Characteristics of STAAB participants.

	Total study sample n = 3901	LV not evaluable n = 972	LV evaluable n = 1831	P (LV evaluable vs not evaluable)	Healthy sample (LV evaluable) n = 479
Age [years]	58 (11)	58 (12)	57 (11)	0.006	52 (10)
Female sex	2033 (52.1)	550 (56.6)	903 (49.3)	< 0.001	270 (56.4)
Body weight [kg]	78 (17)	79 (17)	75 (14)	< 0.001	71 (12)
BMI [kg/m^2^]	26.7 (5.0)	27.0 (5.0)	25.5 (3.8)	< 0.001	23.8 (2.8)
Height [cm]	171 (9)	170 (9)	171 (9)	0.005	172 (9)
BSA [m^2^]	1.9 (0.23)	1.9 (0.23)	1.9 (0.21)	< 0.001	1.8 (0.19)
Syst BP [mmHg]	130 (17)	131 (18)	129 (17)	0.020	121 (10)
Diast. BP [mmHg]	77 (10)	77 (10)	77 (10)	ns	73 (7)
Resting heart rate [1/min]	67 (10)	67 (10)	66 (9)	0.009	65 (8)
Diabetes mellitus^c^	261 (6.7)	63 (6.5)	78 (4.3)	0.014	0 (0)
Dyslipidemia^d^	602 (15.4)	181 (18.6)	214 (11.7)	< 0.001	0 (0)
Hypertension^b^	1915 (49.1)	481 (49.5)	806 (44.0)	0.006	0 (0)
Obesity^a^	775 (19.9)	240 (24.7)	193 (10.5)	< 0.001	0 (0)
CHD^e^	108 (2.8)	29 (3.6)	37 (2.5)	ns	0 (0)
PAD^f^	31 (0.8)	7 (0.7)	10 (0.6)	ns	0 (0)
Stroke^h^	99 (2.5)	25 (2.6)	41 (2.3)	ns	0 (0)
Smoker^g^	1966 (50.4)	502 (51.6)	919 (50.2)	ns	0 (0)
Laboratory values					
Creatinin [mg/dl]	0.9 (0.8, 1.0)	0.9 (0.8, 1.0)	0.9 (0.8, 1.0)	0.003	0.9 (0.8, 1.0)
eGFR [ml/min/1.73m^2^]	81 (15)	81 (15)	82 (14)	ns	85 (14)
Albumin [g/dl]	4.57 (0.27)	4.58 (0.26)	4.58 (0.28)	ns	4.60 (0.26)
Hemoglobin [g/dL]	14.0 (1.2)	14.0 (1.2)	14.0 (1.2)	ns	13.8 (1.2)
Leucozytes [10^9^/L]	5.8 (5.0, 7.0)	5.9 (5.0, 7.0)	5.7 (4.9, 6.8)	0.003	5.5 (4.7, 6.2)
Total cholesterol [mg/dL]	202 (38)	201 (40)	202 (37)	ns	197 (32)
LDL cholesterol [mg/dL]	115 (93, 139)	112 (92, 137)	116 (96, 139)	0.005	114 (94, 136)
HDL cholesterol [mg/dL]	60 (49, 72)	61 (50, 73)	60 (50, 73)	ns	64 (53, 75)
Triglycerides [mg/dL]	97 (71, 140)	97 (70, 141)	92 (69, 133)	0.041	81 (62, 111)
HbA1c [%]	5.55 (0.56)	5.55 (0.52)	5.48 (0.47)	< 0.001	5.30 (0.28)
Fasting BG [mmol/L]	5.2 (1.0)	5.2 (1.0)	5.1 (0.9)	ns	4.9 (0.6)
2D echocardiography values					
LVAVi [mL]	25 (20, 30)	24 (20, 29)	25 (21, 30)	ns	24 (20, 29)
LVMI [g]	74 (18)	74 (19)	73 (18)	ns	66 (16)
LVEDVi [mL]	48 (10)	47 (10)	50 (10)	< 0.001	49 (10)
LV stroke volume [mL]	53 (44, 63)	51 (43, 61)	55 (46, 64)	< 0.001	54 (46, 64)
LV cardiac output [L/min]	3.4 (2.8, 4.1)	3.3 (2.8, 4.0)	3.5 (2.9, 4.1)	0.001	3.4 (2.9, 4.0)
LVEF [%]	59 (5)	58 (5)	60 (5)	< 0.001	61 (4)
E/e′ averaged	7.9 (6.6, 9.7)	7.8 (6.4, 9.4)	7.5 (6.4, 9.2)	0.011	7.0 (6.0, 8.0)
TAPSE [mm]	24 (4)	24 (4)	25 (4)	0.002	25 (3)

Data are given as n (percent), mean (standard deviation) or median (quartiles).

BG: blood glucose, BMI: body mass index, BP: blood pressure, BSA: body surface area, CHD: coronary heart disease, Diast.: diastolic, E: early diastolic mitral inflow velocity, e': early diastolic myocardial velocity, eGFR: estimated glomerular filtration rate, HbA1c: glycated hemoglobin A1c, HDL: high-density lipoprotein, LAVi: left atrial volume index, LDL: low-density lipoprotein, LVEDVi: left ventricular end-diastolic volume index, LV: left ventricular, LVEF: left ventricular ejection fraction, LVMi: left ventricular muscle mass index, PAD: peripheral arterial disease, Syst.: systolic, TAPSE: tricuspid annular plane systolic excursion.

^a^Obesity: BMI > 30 kg/m^2^.

^b^Hypertension: BP ≥ 140/90 mmHg or taking antihypertensive medication.

^c^Diabetes mellitus: HbA1c > 6.5% or taking blood glucose-lowering medication.

^d^Dyslipidemia: LDL-Cholesterol ≥ 190 mg/dl or taking lipid-lowering medication.

^e^Coronary heart disease: self-reported.

^f^Peripheral arterial disease: self-reported.

^g^Smoker: current or former smoker.

^h^Stroke: self-reported.

### Determinants of Twist and Torsion

In the total sample, age was significantly associated with twist (Table 2). Twist increased by + 0.042° per year of age (*p* < 0.001). Sex had a significant effect on twist (+ 1.425° in women, *p* < 0.001). After adjusting for height, this influence was smaller but still significant (+ 1.017° for women, *p* = 0.007). Height and weight were not associated with twist. A significant positive association with age was also shown for torsion (Table 3). Torsion increased by + 0.009°/cm per year of age (*p* < 0.001). A negative relationship was found for height (− 0.007°/cm per cm, *p* = 0.01) and weight (− 0.004°/cm, *p* = 0.009). Sex was significantly associated with torsion (+ 0.297° in women, *p* < 0.001). The association remained significant after adjusting for height (+ 0.221° in women, *p* < 0.001).

**Table 2:** Association of twist (°) with clinical, laboratory and echocardiographic parameters.

	Regression coefficient (95% CI)	*P* value of the regression coefficient	*P* value of sex interaction
Age [per year]*	+ 0.042 (+ 0.018 to + 0.066)	< 0.001	0.825
Sex [female]	+ 1.425 (+ 0.907 to + 1.942)	< 0.001	
Sex [female]**	+ 1.486 (+ 0.969 to + 2.003)	< 0.001	
Sex [female]***	+ 1.017 (+ 0.284 to + 1.751)	0.007	
Height [per cm]	− 0.037 (− 0.011 to − 0.033)	ns	0.754
Body weight [per kg]	− 0.011 (− 0.018 to − 0.067)	ns	0.138
BMI [per kg/m^2^]	− 0.003 (− 0.073 to + 0.068)	ns	0.143
Syst. BP [per mmHg]	+ 0.020 (+ 0.004 to + 0.037)	0.014	0.750
Diast. BP [per mmHg]	+ 0.029 (+ 0.002 to + 0.056)	0.035	0.103
Resting heart rate [per heartbeat]	+ 0.012 (− 0.017 to + 0.040)	ns	0.453
Creatinine [per mg/dL]	+ 0.472 (− 0.631 to + 1.575)	ns	0.504
eGFR [per mL/min/1.73 m^2^]	+ 0.003 (− 0.019 to + 0.024)	ns	0.270
Hemoglobin [per g/dL]	+ 0.022 (− 0.252 to + 0.296)	ns	0.768
HbA1c [per 0.1%]	+ 0.006 (− 0.587 to + 0.598)	ns	0.570
HDL cholesterol [per mg/dL]	+ 0.003 (− 0.014 to + 0.019)	ns	0.727
LDL cholesterol [per mg/dL]	+ 0.001 (− 0.007 to + 0.009)	ns	0.513
Triglycerides [per mg/dL]	+ 0.002 (− 0.016 to + 0.065)	ns	0.749
Fasting BG [per mmol/L]	+ 0.125 (− 0.206 to + 0.457)	ns	0.179
LVEF [per %]	+ 0.252 (+ 0.197 to + 0.306)	< 0.001	0.380
LVEDV [per mL]	+ 0.002 (− 0.011 to + 0.016)	ns	0.397
LVEDVi [per mL]	+ 0.016 (− 0.011 to + 0.044)	ns	0.684
LVM [per g]	− 0.005 (− 0.013 to 0.002)	ns	0.364
LVMi [per g]	− 0.008 (− 0.024 to + 0.009)	ns	0.130
LAVi [per ml]	+ 0.015 (− 0.024 to + 0.053)	ns	0.492
Stroke volume [per mL]	+ 0.037 (+ 0.016 to + 0.077)	< 0.001	0.090
Cardiac output [per L/min]	+ 1.000 (+ 0.000 to + 1.000)	< 0.001	0.098
e′ averaged [per cm/s]	+ 7.109 (− 8.290 to + 22.508)	ns	0.786
E/e′ averaged	+ 0.046 (− 0.084 to + 0.177)	ns	0.654
TAPSE [per mm]			
Women	+ 0.186 (+ 0.079 to + 0.294)	< 0.001	0.023
Men	+ 0.019 (− 0.077 to + 0.116)	ns	
GLS [per %]	+ 0.220 (+ 0.121 to + 0.319)	< 0.001	0.544
GCS 3D [per %]	+ 0.213 (+ 0.159 to + 0.268)	< 0.001	0.200

*Adjusted for sex, **Adjusted for age, ***Adjusted for age and height. All other parameters were adjusted for age and sex.

95% CI: 95% confidence interval, BG: blood glucose, BMI: body mass index, BP: blood pressure, Diast.: diastolic, E: early diastolic mitral inflow velocity, e': early diastolic myocardial velocity, eGFR: estimated glomerular filtration rate, GCS 3D: three-dimensional measured circumferential strain, GLS 3D: three-dimensional measured global longitudinal strain, HbA1c: glycated hemoglobin A1c, HDL: high-density lipoprotein, LAVi: left atrial volume index, LDL: low-density lipoprotein, LV: left ventricular, LVEDV: left ventricular end-diastolic volume, LVEDVi: left ventricular end-diastolic volume index, LVEF: left ventricular ejection fraction, LVM: left ventricular mass, LVMi: left ventricular muscle mass index, ns: not significant, Syst.: systolic, TAPSE: tricuspid annular plane systolic excursion.

**Table 3:** Association of torsion (°/cm) with clinical, laboratory and echocardiographic parameters.

	Regression coefficient (95% CI)	*P* value of the regression coefficient	*P* value of sex interaction
Age [per year]*	+ 0.009 (+ 0.006 to + 0.012)	< 0.001	0.998
Sex [female]	+ 0.297 (+ 0.229 to + 0.364)	< 0.001	
Sex [female]**	+ 0.310 (+ 0.243 to + 0.377)	< 0.001	
Sex [female]***	+ 0.221 (+ 0.126 to + 0.316)	< 0.001	
Height [per cm]	− 0.007 (− 0.012 to − 0.002)	0.010	0.750
Body weight [per kg]	− 0.004 (− 0.007 to − 0.001)	0.009	0.219
BMI [per kg/m^2^]	+ 0.009 (− 0.002 to + 0.016)	ns	0.176
Syst. BP [per mmHg]	+ 0.002 (0.000 to + 0.005)	0.025	0.653
Diast. BP [per mmHg]			
Men	+ 0.007 (+ 0.002 to + 0.012)	0.005	0.044
Women	0.000 (− 0.005 to + 0.005)	ns	
Resting heart rate [per heartbeat]	+ 0.003 (− 0.001 to + 0.006)	ns	0.515
Creatinine [per mg/dL]	+ 0.063 (− 0,080 to + 0.206)	ns	0.663
eGFR [per mL/min/1.73m^2^]	0.000 (− 0.002 to + 0.013)	ns	0.335
Hemoglobin [per g/dL]	+ 0.009 (− 0.029 to + 0.042)	ns	0.518
HbA1c [per 0.1%]	+ 0.009 (− 0.086 to + 0.067)	ns	0.462
HDL cholesterol [per mg/dL]	+ 0.001 (− 0.001 to + 0.003)	ns	0.814
LDL cholesterol [per mg/dL]	0.000 (− 0.001 to + 0.001)	ns	0.339
Triglycerides [per mg/dL]	0.000 ( 0.000 to + 0.001)	ns	0.954
Fasting BG [per mmol/L]	+ 0.014 (− 0.028 to + 0.057)	ns	0.125
LVEF [per %]	+ 0.031 (+ 0.024 bis + 0.038)	< 0.001	0.433
LVEDV [per mL]	− 0.002 (− 0.004 bis − 3.309E−5)	0.046	0.826
LEDVi [per mL]	− 0.001 (− 0.005 bis + 0.002)	ns	0.896
LVM [per g]	− 0.001 (− 0.002 bis − 4.443E−5)	0.041	0.534
LVMi [per g]	− 0.001 (− 0.003 bis + 0.001)	ns	0.367
LAVi [per mL]	0.000 (− 0.005 bis + 0.005)	ns	0.279
Stroke volume [per mL]	+ 0.002 (− 0.001 bis + 0.004)	ns	0.276
Cardiac output [per L/min]	+ 0.052 (+ 0.011 bis + 0.092)	0.012	0.300
E/e′ averaged	+ 0.009 (− 0.008 bis + 0.026)	ns	0.423
e′ averaged [per cm/s]	+ 0.461 (− 1.532 bis + 2.454)	ns	0.907
TAPSE [per mm]			
Women	+ 0.022 (+ 0.008 bis + 0.036)	0.002	0.030
Men	+ 0.001 (− 0.011 bis + 0.014)	ns	
GLS [per %]	+ 0.032 (+ 0.019 bis 0.014)	< 0.001	0.712
GCS 3D [per %]	+ 0.024 (+ 0.018 bis + 0.031)	< 0.001	0.754

*Adjusted for sex, **Adjusted for age, ***Adjusted for age and height. All other parameters were adjusted for age and sex.

95% CI: 95% confidence interval, BG: blood glucose, BMI: body mass index, BP: blood pressure, Diast.: diastolic, E: early diastolic mitral inflow velocity, e': early diastolic myocardial velocity, eGFR: estimated glomerular filtration rate, GCS 3D: three-dimensional measured circumferential strain, GLS 3D: three-dimensional measured global longitudinal strain, HbA1c: glycated hemoglobin A1c, HDL: high-density lipoprotein, LAVi: left atrial volume index, LDL: low-density lipoprotein, LV: left ventricular, LVEDV: left ventricular end-diastolic volume, LVEDVi: left ventricular end-diastolic volume index, LVEF: left ventricular ejection fraction, LVM: left ventricular mass, LVMi: left ventricular muscle mass index, ns: not significant, Syst.: systolic, TAPSE: tricuspid annular plane systolic excursion.

Systolic and diastolic blood pressure showed a positive association with the LV rotational parameters (Tables 2, 3). Twist increased by + 0.020° per mmHg systolic blood pressure (*p* = 0.014) and by + 0.029°/cm per mmHg diastolic blood pressure (*p* = 0.035). Torsion increased by + 0.002°/cm per mmHg systolic blood pressure (*p* = 0.025) and by + 0.007°/cm per mmHg diastolic blood pressure (*p* = 0.005; in men only). Resting heart rate, BMI, and the laboratory parameters estimated glomerular filtration rate, creatinine, albumin, glycosylated hemoglobin, hematocrit, high-density lipoprotein, low-density lipoprotein, triglycerides, and fasting blood glucose showed no association with either twist or torsion (Tables 2, 3).

LVEF, cardiac output, and stroke volume were significantly associated with twist (Table 2). Twist increased by + 0.252° per percent LVEF, by + 1.0° per L/ml cardiac output, and by + 0.037° per ml stroke volume (*p* < 0.001). In women, tricuspid annular plane systolic excursion (TAPSE) was positively associated with twist. Twist increased by + 0.199° per mm TAPSE (*p* < 0.001). Further, there was a numerically inverse association between twist and global longitudinal strain (GLS; + 0.220° per %, *p* < 0.001) and 3D GCS (+ 0.213° per %, *p* < 0.001) independent of sex. Of note, as more negative strain values indicate better function, this translates into a positive association between strain and twist in clinical terms. The parameters LV end-diastolic volume (LVEDV), LVEF, LV mass (LVM), left atrial volume index (LAVi), e' averaged, and E/e′ averaged showed no association with twist.

Torsion also showed a significant positive association with LVEF and cardiac output (Table 3). Torsion increased by + 0.031°/cm per percent LVEF (*p* < 0.001) and by + 0.052°/cm per L/min cardiac output (*p* = 0.012). In women, TAPSE was positively associated with torsion (+ 0.022°/cm per mm TAPSE, *p* = 0.002). GLS and GCS were associated with torsion independently of sex. Torsion increased by + 0.032°/cm per percent GLS (*p* < 0.001) and by + 0.024°/cm per percent GCS (*p* < 0.001). In addition, a negative association with LVEDV and LVM was found for torsion. Torsion decreased by -0.002°/cm per ml LVEDV (*p* = 0.046) and by − 0.001°/cm per g LVM (*p* = 0.041). No association was found for the indexed parameters LVEDVi and LVMi. Likewise, no association could be found for the parameters stroke volume, LAVi, e' averaged and E/e′ averaged.

## Reference values

In apparently healthy individuals, median (95% confidence interval) twist was 10.72° (1.9°; 23.02°) and median torsion was 1.37°/cm (0.26°/cm; 3.06°/cm). There we found a significant association of torsion with age and height. Hence, we determined reference values for twist as well as age- and height-adjusted reference values for torsion. For any age and height, reference percentiles for torsion can be calculated with the following formula:

$$\begin{aligned} & {\text{Constant}}\,{\text{term}} + (deviation\,in\,cm\,from\,height\,170\,cm)*{\text{B}}\,{\text{h}}170 \, \\ & \quad + (deviation\,in\,years\,from\,age\,50\,years)*{\text{B}}\,{\text{a}}50 + {\text{percentile}}\,{\text{X}} \\ \end{aligned}$$

Abbreviations and the percentiles for twist and torsion are presented in Table 4.

**Table 4:** Reference percentiles of 3D echocardiography-derived twist and torsion.

	Torsion (°/cm)	Twist (°)
B h170 (Sig.)	− 0.011 (*p* = 0.021)	Ns
B a50 (Sig.)	+ 0.007 (*p* = 0.042)	Ns
B Men	ns	Ns
Constant term	+ 1.464	+ 11.130
Percentiles		
2.5	− 1.20	− 9.23
10	− 0.81	− 6.53
25	− 0.48	− 3.74
50	− 0.09	− 0.41
75	+ 0.42	+ 3.40
90	+ 0.93	+ 7.18
97.5	+ 1.60	+ 11.89

There was no interaction between sex and height or sex and age for twist or torsion.

a50: covariate that sets the zero point of the constant term to age 50, B: regression coefficient, h170: covariate that sets the zero point of the constant term to body height 170 cm, ns: not significant.

Exemplary calculation of the median torsion (i.e., 50^th^ percentile) for a person with body height 172 cm, aged 64 years:

1.464°/cm + 2*(− 0.011°/cm) + 14*(0.007°/cm) +  ( − 0.09°/cm) = 1.45°/cm.

*[Constant term* + (deviation in cm from height 170 cm)*B h170 + (deviation in years from age 50 years)*B a50 + *deviation in 50*^*th*^* percentile].*

Exemplary calculation of the median twist of the same person:

11.13° + (− 0.41°) = 10.72°

*[Constant term* + *deviation in 50th percentile].*

## Discussion

To our knowledge, we here provide the first analysis regarding determinants of LV twist and torsion derived from a well characterized population-based sample stratified for age and sex. We found a concordant association of LV rotational parameters with other parameters of systolic LV function like LVEF, longitudinal and circumferential strain as well as stroke volume and cardiac output. From a carefully selected subgroup of healthy individuals, we derived reference values for LV twist and torsion. Because LV torsion was related to age and height, the corresponding normal values were provided in strata for age and height.

In the total sample, we found LV rotational parameters further associated with sex, suggesting a potential sex-specific effect of cardiovascular risk factors on LV rotation. Higher blood pressure was associated with higher LV twist and torsion, potentially mediated by increased forces of the epicardial fibers, whereas higher body weight was associated with lower LV torsion, which might be the consequence of weakened epicardial fibers. Further research is needed to confirm our findings and to extend them by longitudinal data.

### Physiology of LV rotation

Physiologically, the myocardium shortens longitudinally and circularly during systole. Simultaneously, there is a twisting of the LV around its longitudinal axis^25^. A complex arrangement of fibers in the layers of the myocardium underlies this contraction pattern. These fibers form a right-handed helix in the inner subendocardial layer and run circularly across the middle layer to a left-handed helix in the outer subepicardial layer (Fig. 1). This spiral arrangement of fibers results in an opposite rotation of the cardiac apex and base during systole^26^.

The counteraction of the subepi- and subendocardial fibers is crucial for the ultimate extent of the twist. In general, the subepicardial fibers generate a greater force because of the longer axis of rotation. If there is an additional increase in force of the already dominant subepicardial fibers, the twist increases. On the other hand, any factor that weakens the subendocardial fibers can also lead to an increase in twist^12^.

### Methodological aspects

To date, only limited data has been published on reference values for LV twist and torsion based on a large sample. The *World Alliance Societies of Echocardiography* (WASE) study ^15^ recruited healthy volunteers from 15 countries worldwide including different ethnicities, aiming for an even distribution of age and sex. We provide an analysis that further extends these results being based on a well-characterized population-based sample that has been recruited strictly stratified for age and sex, a methodology to minimize a potential sampling error, and which further makes it possible both to generate reference values and to identify determinants of rotational parameters from the same population. The feasibility in the healthy reference population in our study was 73.5%, which is comparable to WASE, which reached a feasibility of 70% for the 3D measurement of twist and torsion^15^, highlighting once more the challenge to acquire proper 3D images from the transthoracic window.

### Determinants of twist and torsion

#### Age

Our study showed a significant positive association of LV twist and torsion with age, both, in the total sample as well as in the sub-collective of healthy participants. Older subjects had higher values for twist and torsion than younger subjects. This result is consistent with the findings of a longitudinal MRI study ^11^ and echocardiography studies in smaller samples that also used 3DE as a measurement method ^9,12,13^. The finding may be explained by progressive remodeling and weakening of subendocardial fibers with age which is associated with a predominant reduction in subendocardial LV function^27–29^. The reduction in rotational forces in the subendocardium leads to an increase in rotation induced by the already dominant subepicardial fibers and thus to an increase in twist^30^. An increase in rotational deformation could be a compensatory mechanism to maintain systolic function with increasing age.

#### Sex

In our healthy sub-collective, there was no association of LV rotational parameters with sex. In contrast, in the total collective with evaluable LV, there was a significant association of LV rotational parameters with sex, which remained significant after several adjustments. Women consistently showed higher values for twist and torsion when compared to men. An MRI study also showed higher torsion in women^10^. Yoneyama et al. also describe negative associations of torsion with EDV and ESV and suggest that in smaller hearts, and therefore in women, higher torsion is required to maintain adequate cardiac output^10^. Williams et al. showed a higher twist in women under acute reduction of preload to maintain mean arterial pressure^31^. Another study found that LV twist mechanics are more sensitive to changes in adrenergic stimulation in men, whereas they are more influenced by ventricular structure and geometry in women^32^. It has been shown that women have a more ellipsoid-shaped ventricle with a higher sparsity index^31^. A sparse LV shape entails a horizontal fiber arrangement, which promotes higher torsion^10^.

Further research is needed to elucidate the mechanisms behind the observed higher rotational deformation in women. Nevertheless, the fact, that the association of LV rotational parameters with sex was evident in the total collective but not in the healthy sub-collective might also suggest, that cardiovascular risk factors might have a sex-specific impact on LV rotation.

#### Height and bodyweight

Body height showed no association with twist, neither in the healthy sub-collective nor in the total sample. Nevertheless, the indexed parameter torsion showed a negative association with body height in both collectives. This might be explained by a certain amount of twist required to maintain adequate cardiac output, consecutively resulting in lower torsion (= twist/LV length) in larger hearts^10^. On the other hand, we found a negative association of LV torsion with body weight. These findings are consistent with a study that associated a depressed GLS with an increased afterload in obese persons and found that GLS recovered following bariatric surgery^33^. Increased body weight and obesity might contribute to changes in LV geometry and LV afterload resulting in a decrease in the force of the epicardial fibers, which, in turn, would result in lower twist and torsion.

#### Blood pressure

Several studies have investigated the relationship between arterial hypertension and torsion^10,11,34^. Both MRI^10,11^ and 3DE^34^ showed increased values for torsion in subjects with arterial hypertension. Cameli et al. who examined arterial hypertension in a stage-dependent manner for association with torsion, found an increase in torsion in the early stages of hypertension and a decrease in the advanced stage with eccentric hypertrophy^35^. In our study, there was a positive association between both twist and torsion and systolic and diastolic blood pressure, the latter only in men. In the early stages of hypertension with endocardial fiber impairment, an increase in torsion could be a compensatory mechanism to maintain systolic function.

#### Parameters of cardiac function

Previous studies have shown a positive association between rotational parameters and LVEF^34,36,37^. This association could be confirmed in our data. Stuber et al. showed increased torsion in subjects with aortic valve stenosis and preserved LVEF compared to controls^38^. In other studies, increased torsion was found in subjects with hypertension with preserved LVEF, while the longitudinal strain and in one study also the circumferential strain were reduced^34,39^. Minatoguchi et al. found preserved torsion in heart failure with preserved ejection fraction, whereas it was reduced in heart failure with reduced ejection fraction^34^.

LV rotational deformation appears to be a critical factor in maintaining LVEF and systolic function in the face of factors that impair subendocardial function. The positive association between twist/ torsion and stroke volume and cardiac output found in our study supports this assumption. An MRI study demonstrated a decrease in torsion with increasing LV mass and increasing LVEDV^11^. These associations could be confirmed in our data using 3DE. The observation of Yoneyama et al. that larger hearts have lower mean values for torsion may play a role here^10^. Yoneyama et al. also suggested that fibrosis of the myocardium observed in hypertrophy in response to pressure overload due to collagen deposition^40^ could explain the negative association between torsion and LVM^10^. In our study, there was further an inverse, hence concordant association between the parameters of rotational deformation and those of longitudinal and circumferential deformation. This suggests a physiological coupling of the deformation parameters.

To the best of our knowledge, we are the first study describing an association of twist/ torsion and TAPSE. TAPSE is a useful parameter to assess right ventricular function. A decrease of TAPSE (< 1.6 cm) indicates a limitation of right ventricular function^41^. According to our results, decreased LV rotation in women is related to decreased TAPSE. Damiano et al. showed that geometric deformations of the LV can have significant mechanical effects on the RV. The authors explained this with the deformation of the RV free wall, which is interconnected with the LV via the interventricular septum^42^, hence, LV structure and function of the LV inadvertently affect the RV. Hoffman et al. also demonstrated a significant influence of LV contraction on RV function^43^. These findings as well as the fact, that LV and RV function share certain risk factors, may explain the association of TAPSE with LV twist and torsion found in our study. As described above, LV rotation might be more sensitive to changes in adrenergic stimulation in men, but more influenced by ventricular structure and geometry in women^32^. This might be a potential explanation for our finding that TAPSE was associated with LV rotation only in women, but this hypothesis deserves further investigation in larger cohorts.

### Limitations

Some limitations need to be considered when interpreting our results. Our study includes a representative sample of the city of Würzburg and thus predominantly Caucasian subjects. This might limit the application of our results to other populations. Only one echocardiographic 3D examination was performed per subject, which is why we cannot make any statements about test–retest reliability. Further, measurement of twist and torsion might differ when using software of another vendor. Future research should examine an intervendor variability for the measurement of 3D-derived twist and torsion and validate our results. Still, our findings rely on a robust methodology that involves the utilization of standardized echocardiograms from a well-characterized population-based cohort.

## Conclusions

LV rotation is determined by a complex interplay of sub-endocardial and sub-epicardial fibers which might be affected differentially by potential risk factors. We found a differential association with respective determinants as LV rotational parameters increased with age and with higher blood pressure but decreased with higher body weight. Further research is needed to elucidate these associations in more detail and to determine the additional information contributed by LV rotational parameters. To facilitate respective attempts and to set individual results in relation to a population-based reference, we derived normal values for twist and torsion from a sub-collective of healthy individuals.

## Clinical perspective

An integration of the measurement of twist and torsion parameters into clinical routine might offer great potential owing to their previously shown prognostic relevance they provide.Measuring the rotational function parameters could be used to detect the severity of conditions that in particular affect the cardiac subendothelial function at early stages, and might be used as a follow-up parameter to monitor disease progression or triggering preventive therapeutic measures. Longitudinal assessment in our and similar cohorts might reveal the course of twist and torsion in healthy ageing as well as in the development of cardiac diseases like hypertensive or diabetic heart disease, potentially resulting in heart failure with preserved ejection fraction.

In addition, routine co-recording of rotational parameters could add to the interpretation of LVEF. In cardiac diseases, especially those progressing towards heart failure with preserved ejection fraction like storage diseases or cardiotoxic exposure, LVEF remains compensated for a long time despite substantial cardiac alteration. A worsening in longitudinal function is considered a first, sensitive sign of myocardial impairment. In turn, longitudinal function is the results of concurring forces of the subendocardial and epicardial fibers, whose function is sensitively captured by LV twist and torsion. Thus, by combining LVEF, longitudinal strain, and rotational parameters, an improved approach for detection, classification, and risk assessment of heart disease might be developed.

## Supplementary Information

Supplementary Information 1.

Supplementary Information 2.

Supplementary Information 3.

## Data Availability

Data and materials are available on request from the corresponding author.

## Abbreviations

3D: Three-dimensional
3DE: Three-dimensional echocardiography
BMI: Body mass index
DCM: Dilated cardiomyopathy
E: Early mitral inflow velocity
e′: Mitral annulus relaxation velocity
eGFR: Estimated glomerular filtration rate
GCS: Global circumferential strain
GLS: Global longitudinal strain
Hb: Hemoglobin
HDL: High-density lipoprotein
LAVi: Left atrial volume index
LDL: Low-density lipoprotein
LV: Left ventricle
LVEDV: Left ventricular end-diastolic volume
LVEF: Left ventricular ejection fraction
LVESV: Left ventricular end-systolic volume
LVM: Left ventricular mass
MRI: Magnetic resonance imaging
RV: Right ventricle
STAAB: Characteristics and Course of Heart Failure Stages A-B and Determinants of Progression Cohort Study
TAPSE: Tricuspid annular plane systolic excursion
WASE: World Alliance Societies of Echocardiography
