# Supplementary material for: 3D echocardiography derived reference values and determinants of left ventricular twist and torsion from the population-based STAAB cohort study
Source: Sci Rep. 2025 Feb 6;15:4524. doi: 10.1038/s41598-024-81662-x (PMC11802741; doi:10.1038/s41598-024-81662-x)
Supplement: Supplementary file 1 — Supplementary Information 1. [file 41598_2024_81662_MOESM1_ESM.pdf]

Additional figure 1: Interobserver variability for 3D echocardiography-derived left ventricular twist (left) and torsion (right).

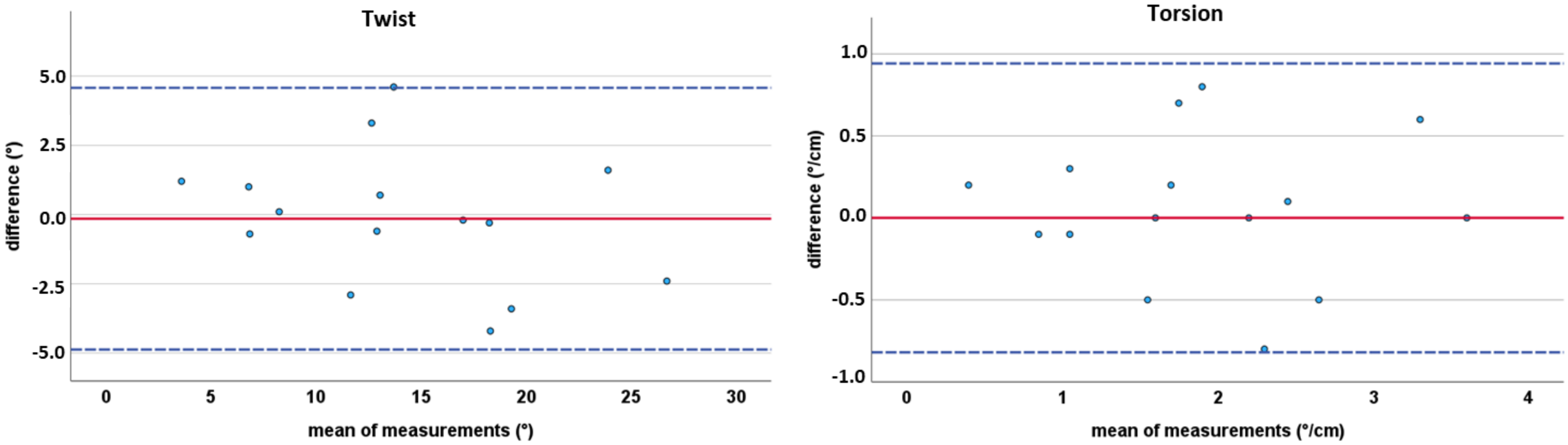

Bland-Altman plots. X-axis: mean of measurements in ° (left) and °/cm (right). Y-axis: the difference between measurements between observer 1 and 2 in ° (left) and °/cm (right).
