# Supplementary material for: 3D echocardiography derived reference values and determinants of left ventricular twist and torsion from the population-based STAAB cohort study
Source: Sci Rep. 2025 Feb 6;15:4524. doi: 10.1038/s41598-024-81662-x (PMC11802741; doi:10.1038/s41598-024-81662-x)
Supplement: Supplementary file 3 — Supplementary Information 3. [file 41598_2024_81662_MOESM3_ESM.docx]

**Additional table 1:** 3D echocardiography-derived parameters of the LV

|  | Total  sample  (LV evaluable) | Women | Men | P-value (women  vs men) | Subset of apparently healthy persons | Women | Men | P-value (women  vs men) |
| --- | --- | --- | --- | --- | --- | --- | --- | --- |
| N [%] | 1831 (100) | 903 (49.3) | 928 (50.7) |  | 479 (26.2) | 270 (14.7) | 209 (11.4) |  |
| LVEF [%] | 61 (7) | 62 (7) | 59 (6) | <0.001 | 60 (6) | 61 (5) | 58 (6) | <0.001 |
| LVEDV [ml] | 101 (25) | 89 (17) | 114 (26) | <0.001 | 100 (25) | 88 (17) | 115 (25) | <0.001 |
| LVEDVi [ml/m²] | 54 (11) | 51 (9) | 57 (12) | <0.001 | 54 (11) | 51 (9) | 59 (12) | <0.001 |
| LVESV [ml] | 38 (31, 47) | 34 (27, 39) | 45 (37, 54) | <0.001 | 39 (32, 47) | 34 (28, 40) | 46 (39, 57) | <0.001 |
| LVESVi [ml/m²] | 20 (17, 24) | 19 (16, 22) | 22 (19, 27) | <0.001 | 21 (18, 25) | 20 (17, 23) | 24 (20, 28) | <0.001 |
| GLS [%] | -21.7 (4) | -22.3 (4) | -21.0 (4) | <0.001 | -21.6 (4) | -22.2 (4) | -20.8 (4) | <0.001 |
| GCS [%] | -28.7 (5) | -29.3 (5) | -28.0 (5) | <0.001 | -28.4 (4) | -28.9 (4) | -27.8 (4) | <0.001 |

Data are given as n (percent), mean (standard deviation) or median (quartiles).

GCS: global circumferential strain, GLS: global longitudinal strain, LVEDV: left ventricular end-diastolic volume, LVEDVi: left ventricular end-diastolic volume index, LVEF: left ventricular ejection fraction.
